# Effect of *Lactobacillus rhamnosus* AB-GG combined with phototherapy on neonatal jaundice indicators, intestinal microbiota and metabolism

**DOI:** 10.3389/fnut.2025.1581242

**Published:** 2025-04-08

**Authors:** Yanhan Yuan, Jiayi Chen, Tianyu Liu, Juanjuan Chen, Feng Zhang, Zhe Shi, Jinping Zhang

**Affiliations:** ^1^Department of Pediatrics, Shanghai Sixth People’s Hospital Affiliated to Shanghai Jiao Tong University School of Medicine, Shanghai, China; ^2^College of Food Science and Technology, Shanghai Ocean University, Shanghai, China

**Keywords:** jaundice, phototherapy, gut microbiota, *Lactobacillus rhamnosus*, neonates

## Abstract

**Objectives:**

To assess whether *Lactobacillus rhamnosus* AB-GG supplementation has a treatment effect on the neonatal jaundice of infants receiving phototherapy.

**Methods:**

In this study, 11 and 10 neonates in experimental and control groups were recruited, respectively (upon the follow-up of experimental groups at 7–14 days after discharge, stool frequency was decreased commonly. Therefore, this study was prematurely terminated). After 12 h of phototherapy, researchers recorded clinical information and measured transcutaneous bilirubin (TCB). Fresh fecal samples were collected at seven specific time points: before phototherapy (A), after 12 (B), 24 (C) and 36 h (D) of phototherapy, as well as 10 (D10), 20 (D20) and 30 days (D30) after delivery.

**Results:**

A tendency toward fewer blue light sessions and shorter time of hospitalization was shown in experimental groups, but this exhibited no statistical significance (*p* > 0.05). Compared with the experimental groups, phototherapy significantly reduced the alpha (α) diversity of intestinal flora in the control groups. However, phototherapy had no significant effect on beta (β) diversity between experimental and control groups. This study also observed that the metabolic composition structures of both groups underwent changes before and after phototherapy. However, no significantly differential metabolites were identified when the groups were compared at different time points.

**Conclusion:**

*Lactobacillus rhamnosus* supplementation was shown to mitigate intestinal dysbiosis in jaundiced neonates, which thereby facilitated a more rapid recovery of gut microbiota depleted by phototherapy.

**Clinical trial register:**

It was registered with the Chinese Clinical Trial Registry (Registration No.: ChiCTR2000036013).

## 1 Introduction

Characterized by the elevation of serum bilirubin, neonatal jaundice results in the yellow discoloration of the skin, sclera and mucous membranes. It is divided into physiologic and pathologic jaundice (1). Pathologic neonatal jaundice is the most common cause of hospitalization during the neonatal period (2). The gut microbiota participates in the metabolism of bilirubin through two primary pathways. The gut microbiota has an important role in mediating the transformation of conjugated bilirubin to unconjugated bilirubin, and then unconjugated bilirubin is further turned into bilinogen to be excreted out of the body. Also, the gut microbiota can alters intestinal osmotic pressure and pH, inhibits the hydrolysis of conjugated bilirubin by β-glucuronidase, stimulates intestinal motility, and accelerates the excretion of bilirubin through interaction among different microbial species or their metabolic byproduct (3, 4). At present, phototherapy is regarded as the most frequently used, safe and useful method of reducing the level of serum bilirubin, which thereby diminishes the occurrence of severe hyperbilirubinemia and bilirubin encephalopathy (5). However, phototherapy is related to rash, fever, diarrhea and other adverse effects (6). Preliminary research suggests that these side effects may be correlated with phototherapy-induced gut microbiota disturbances, with significant reductions of probiotic strains in the gut microbiota observed (7). Studies have also demonstrated that the administration of probiotics can mitigate these adverse effects, enhance the efficacy of phototherapy, and potentially reduce the risk of bilirubin encephalopathy (8, 9). Nevertheless, no consensus has been reached on the selection of probiotic strains during phototherapy for neonatal jaundice. Based on previous findings, *Lactobacillus rhamnosus* AB-GG, a strain significantly decreased during phototherapy, was incorporated in this study to observe its impact on clinical outcomes and gut microbiota and metabolism in jaundiced neonates undergoing phototherapy (10, 11). This approach was aimed at theoretically supporting the rational application of probiotics during phototherapy for neonatal jaundice.

## 2 Methods

### 2.1 Study design

This is a single-center randomized controlled clinical trial conducted at the Shanghai Sixth People’s Hospital affiliated to Shanghai Jiao Tong University School of Medicine (SJTUSM). It was registered with the Chinese Clinical Trial Registry (Registration No.: ChiCTR2000036013). Digital random allocation was used as the randomized controlled approach.

This study gained the approval of the Ethics Committee of the East Hospital of Shanghai Sixth People’s Hospital according to relevant regulations like the Declaration of Helsinki (Ethics Approval No.: 2020–071). An informed consent form was signed by all enrolled neonatal parents.

### 2.2 Participants

The inclusion criteria included (1) Jaundice index: It reached the threshold for phototherapy in accordance with the phototherapy guidelines of the 2022 American Academy of Pediatrics; (2) Postnatal age: ≤2 weeks; (3) Full-term infants: Gestational age ≥ 37 weeks and <42 weeks; birth weight ≥ 2,500 g and <4,000 g; (4) Specimen collection: No antibiotics or probiotics were administered before sample collection; (5) Maternal health during pregnancy: the mother was in good health without any notable medication history, and no antibiotics or probiotics were taken during prepartum, intrapartum or postpartum periods; (6) Informed consent: An informed consent form was voluntarily signed.

The exclusion criteria included (1) Gestational age ≥ 42 weeks or <37 weeks; (2) Bilirubin levels reached the standard for exchange blood transfusion or elevated direct bilirubin; (3) Patients concomitantly diagnosed with diseases like pneumonia and septicemia; (4) Patients developing severe immunodeficiency; (5) Patients with genetic metabolic disorders; (6) Cases with organ anomalies like congenital biliary abnormalities; (7) Documented drug hypersensitivity scenarios; (8) Individuals at risk of compromising adherence to the study based on the judgment of the investigator, like guardians with psychiatric conditions or frequent changes in the living or working environment, and thus having escalated chances for non-compliance with the study.

### 2.3 Sample size

The rate of side effects of phototherapy is approximately 47% based on prior research. In our study, we aim to reduce this incidence to around 20% through the application of probiotics. Margin, type I error and power were assumed to be 0.05, 0.05 and 0.8, respectively. A difference test was used for two-sample ratios, and a sample size of 18 cases in each group was computed, which resulted in 36 cases in experimental and control groups.

### 2.4 Intervention

All participants were hospitalized between August 2021 and March 2022 at the Neonatal Department of Lingang Campus of the Shanghai Sixth People’s Hospital attached to SJTUSM. Upon the follow-up of the experimental groups at 7–14 days after discharge, stool frequency was decreased commonly. About 3 days after stopping probiotics, stool frequency is normal in the experimental groups. However, this study was still prematurely terminated. In this study, 11 and 10 neonates in experimental (T3 group) and control groups (C group) were included, respectively.

The dose of probiotics (*L. rhamnosus* AB-GG) was 10^9^ colony-forming units that were administered orally once every day for 1 month. After 12 h of phototherapy, researchers recorded clinical information and measured transcutaneous bilirubin (TCB). Fresh fecal samples were collected at seven specific time points: before phototherapy (A), after 12 (B), 24 (D) and 36 h (D) of phototherapy, as well as 10 (D10), 20 (D20) and 30 days (D30) after delivery.

### 2.5 Stool sample

Fresh stool samples were collected from each subject and were stored within 30 min in a −80°C freezer during hospital stay. After discharge, tool samples were collected in sterile plastic containers and stored within 30 min in a home freezer at −20°C. Following collection, these samples were transferred to a −80°C freezer within 24 h. All stool samples were stored in a −80°C freezer until processed.

### 2.6 Metagenomic profiling

#### 2.6.1 Deoxyribonucleic acid extraction and sequencing

An ultrasonic crusher was utilized to randomly break the qualified deoxyribonucleic acid (DNA) samples into fragments of around 350 bp in length. The whole library preparation was completed via end repair, the addition of 3′ end A and sequencing adapters, purification, fragment selection, polymerase chain reaction (PCR) amplification and other steps. After the completion of library construction, the quantitative PCR (qPCR) method was employed to accurately quantify the effective concentration of the library (library effective concentration > 3 nM) to ensure its quality for further sequencing. A 2 × 150 bp paired-end protocol was used to sequence metagenomic DNA on Illumina HiSeq.

#### 2.6.2 Sequencing data quality control

Trimmomatic (v_0.39) was applied to remove low-quality sequences. The quality control of sequencing reads was conducted to remove low-quality reads and trim low-quality bases. KneadData was adopted to remove the contamination sequence from human DNA. Before and after removal, FastqQC was used to examine sequence quality.

#### 2.6.3 Taxonomy annotation

Host-filtered microbial reads underwent classification against viral, bacterial, archaeal, fungal and human genomes by use of Kraken2 on a reference database. With bacteria, fungi, archaea and virus sequences, the reference database was constructed from NCBI nucleotide and RefSeq database. After that, the classification report was used by Bracken for estimating species abundance, which provided estimated reads per species.

#### 2.6.4 Functional annotation

Functional analysis was made using HUMAnN2 based on the UniRef90 database and annotated by the Kyoto Encyclopedia of Genes and Genomes (KEGG) database to get KEGG ontology (KO) and pathway level profile per sample.

### 2.7 Metabolite profiling

#### 2.7.1 Sample preparation

Liquid nitrogen was leveraged to ground feces (100 mg) individually, and prechilled 80% methanol was used to resuspend the homogenate by well vortex. The samples were subjected to 5-min incubation on ice and then 20-min centrifugation at 15,000 *g* at the temperature of 4°C. Liquid chromatography-mass spectrometry (LC–MS) grade water was utilized to dilute some of the supernatants to the final concentration with 53% methanol, followed by the transfer of the samples to a fresh Eppendorf tube and their 20-min centrifugation at 15,000 *g* at 4°C. At last, the LC–MS/MS system was injected with the supernatant for analysis.

#### 2.7.2 LC–MS analyses

A Vanquish UHPLC system (Thermo Fisher, Germany), along with an Orbitrap Q Exactive™ HF or Orbitrap Q Exactive™ HF-X mass spectrometer (Thermo Fisher, Germany) was used to perform ultra-high performance liquid chromatography (UHPLC)-MS/MS analyses. A 17-min linear gradient was utilized to inject the samples onto a Hypersil GOLD column (100 × 2.1 mm, 1.9 μm) at a flow rate of 0.2 mL/min. The eluents for positive and negative polarity modes were eluents A (0.1% FA in water) and B (methanol), and eluents A (5 mM ammonium acetate, pH 9.0) and B (methanol), respectively. Below was the set solvent gradient: 2% B, 1.5 min; 2–85% B, 3 min; 85–100% B, 10 min; 100–2% B, 10.1 min; 2% B, 12 min. Q Exactive™ HF mass spectrometer was operated in the positive/negative polarity mode. The spray voltage was 3.5 kV; the capillary temperature was 320°C; the sheath gas flow rate was 35 psi; the aux gas flow rate was 10 L/min; the S-lens RF level was 60; the Aux gas heater temperature was 350°C.

#### 2.7.3 Data processing and metabolite identification

Compound Discoverer 3.3 (CD3.3, Thermo Fisher) was adopted to process the raw data files produced by UHPLC–MS/MS to align and pick peaks for and quantify each metabolite. Below were the set main parameters: peak area corrected with the first QC, an actual mass tolerance of 5 ppm, a signal intensity tolerance of 30% and minimum intensity. Next, the precise qualitative and relative quantitative results were obtained by matching peaks with the mzCloud^1^, mzVault and MassList database, and the KEGG database was employed to annotate metabolites^2^. Peak intensities were normalized with the median value per sample, log-transformed and normalized with mean and standard deviation per metabolite. The normalized data were used for further analyses including principal component (PCA), differential and correlation analyses with species abundance. The statistical software R (R version R-4.3.1) was applied to perform statistical analyses.

### 2.8 Bioinformatic analysis and statistics

Student’s *T*-test was used for analyzing the alpha (α) diversity between groups, and Bray–Curtis dissimilarity was employed to calculate beta (β) diversity. The impact of phenotype on taxon/metabolite profiles was evaluated by performing permutational multivariate analysis of variance (PERMANOVA) utilizing the “adonis” function in the R Vegan package.

## 3 Results

### 3.1 Clinical data

#### 3.1.1 Subject characteristics

Basal characteristics of the experimental and control groups were shown in Table 1. Both groups were not significantly different in terms of gestational age, age, weight, gender, delivery mode and feeding method (*p* > 0.01) (Table 1).

**Table 1:** Characteristics of oral data of different groups.

Parameters	Control (*n* = 10)	Experimental (*n* = 11)	*P*-value
Gestational age (day)	268.4 ± 8.8	270.1 ± 10.3	0.692
Age (day)	4.7 ± 2.5	3.9 ± 2.0	0.423
Birth weight (g)	3331.0 ± 447.5	3,256.4 ± 382.8	0.685
Gender			
Males, *n*(%)	4 (40)	3 (27.3)	0.537
Females, *n*(%)	6 (60)	8 (72.7)	
Mode of delivery			
Natural birth, *n*(%)	5 (50)	7 (63.6)	0.528
Cesarean section, *n*(%)	5 (50)	4 (36.4)	
Feeding			
Breast feeding, *n*(%)	2 (20)	1 (9.1)	0.096
Formula, *n*(%)	7 (70)	4 (36.4)	
Mix, *n*(%)	1 (10)	6 (54.5)	

#### 3.1.2 Clinical outcomes

The two groups showed no significant differences in changes in body weight, daily defecation frequency, the degree of decrease in pre- and post-treatment transcutaneous bilirubin levels, the number of times blue light therapy was administered during hospitalization and the length of hospital stay (Table 2). Specifically, a tendency toward fewer blue light sessions and shorter time of hospitalization was shown in experimental groups, but it exhibited no statistical significance compared with the control group (*p* > 0.05).

**Table 2:** Clinical outcomes of oral data of different groups.

Parameters	Control	Experimental	*P*-value
Patient number	10	11	
Weight change (%)	−1.51 ± 6.42	−0.65 ± 7.12	0.76
Fecal times per day	3.5 ± 1.08	3.7 ± 1.33	0.70
Post-treatment skin bilirubin (mg/dl)	16.05 ± 2.99	14.07 ± 2.35	0.10
Skin bilirubin loss ratio (%)	62.24 ± 16.08	62.01 ± 19.44	0.97
Phototherapy number (12 h each time)	2.83 ± 1.03	2.00 ± 0.94	0.06
Days in hospital	4.33 ± 1.96	3.50 ± 1.08	0.83

### 3.2 Gut microbiota

#### 3.2.1 Exchange of operational taxonomic units

**Figure 1:**
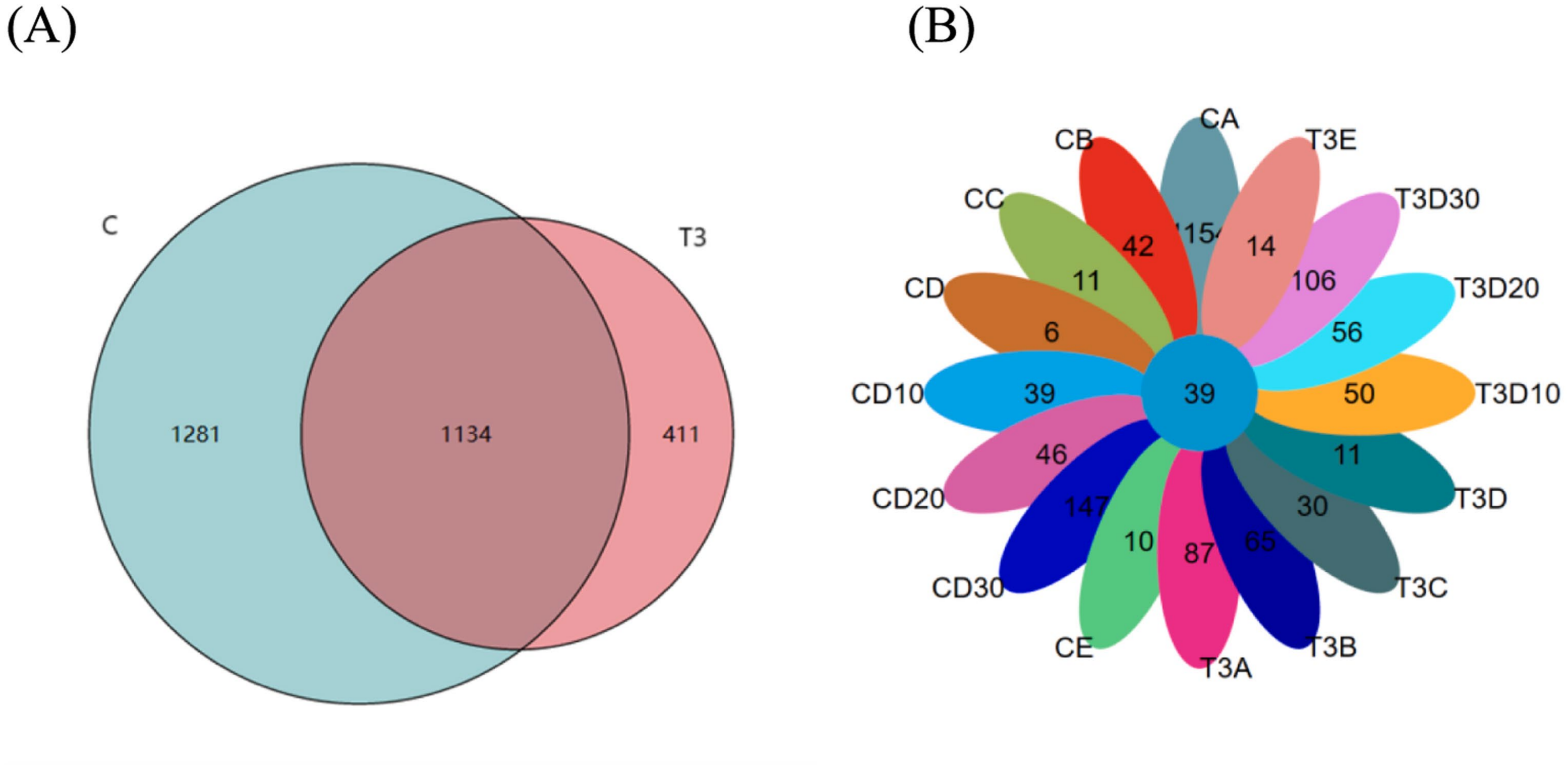
**(A)** All OTUs in experimental and control groups. **(B)** Experimental and control groups in different sampling periods of OTUs.

In the present study, 2,826 operational taxonomic units (OTUs) were identified. The experimental group was composed of 1,545 OTUs, while the control one consisted of 2,415 OTUs, with 1,134 OTUs common to both groups (Figure 1A). The OTU count in the control group peaked before phototherapy, whereas that in the experimental group reached the peak on the 30th day of follow-up. Experimental and control groups both demonstrated a marked decline in the number of OTUs during phototherapy, with the number of OTUs recovering to a greater extent as post-phototherapy duration lengthened. After the follow-up, the control group showed a decrease of 1,007 OTUs compared to pre-phototherapy levels, whereas the experimental one registered an increase of 19 OTUs (Figure 1B).

#### 3.2.2 α diversity

**Figure 2:**
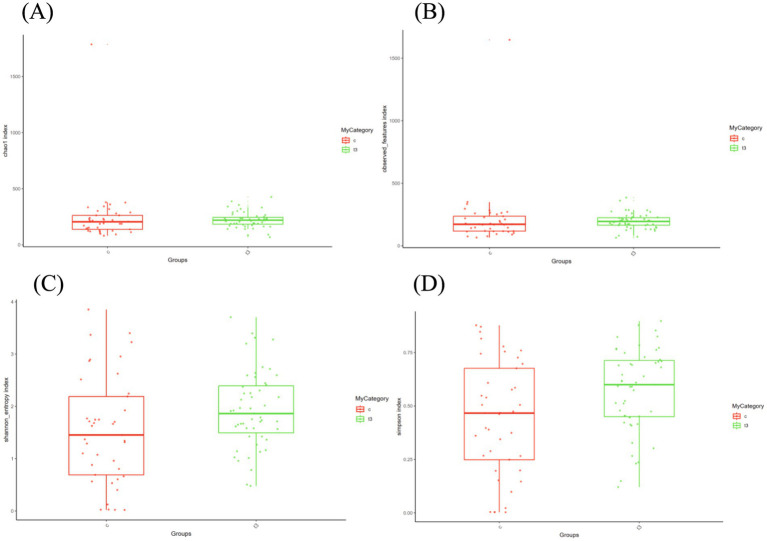
**(A)** Chao1 indices in experimental and control groups. **(B)** Observed indices in experimental and control groups. **(C)** Shannon indices in experimental and control groups. **(D)** Simpson indices in experimental and control groups.

In the current study, α diversity analysis reflecting the richness and evenness of a specific ecosystem was employed with Observed and Chao1 indices to evaluate richness, and Shannon and Simpson indices to assess the evenness of the microbial community. The experimental group showed elevated Chao1 (*p* = 0.012) and Observed indices (*p* = 0.041) compared with the control one, which indicated a tendency for increased species richness in the experimental group (Figures 2A,B). Similarly, the experimental group had higher Shannon (*p* = 0.028) and Simpson indices (*p* = 0.033) than the control one, which signified greater evenness in the microbial community of the experimental group (Figures 2C,D).

#### 3.2.3 β diversity

**Figure 3:**
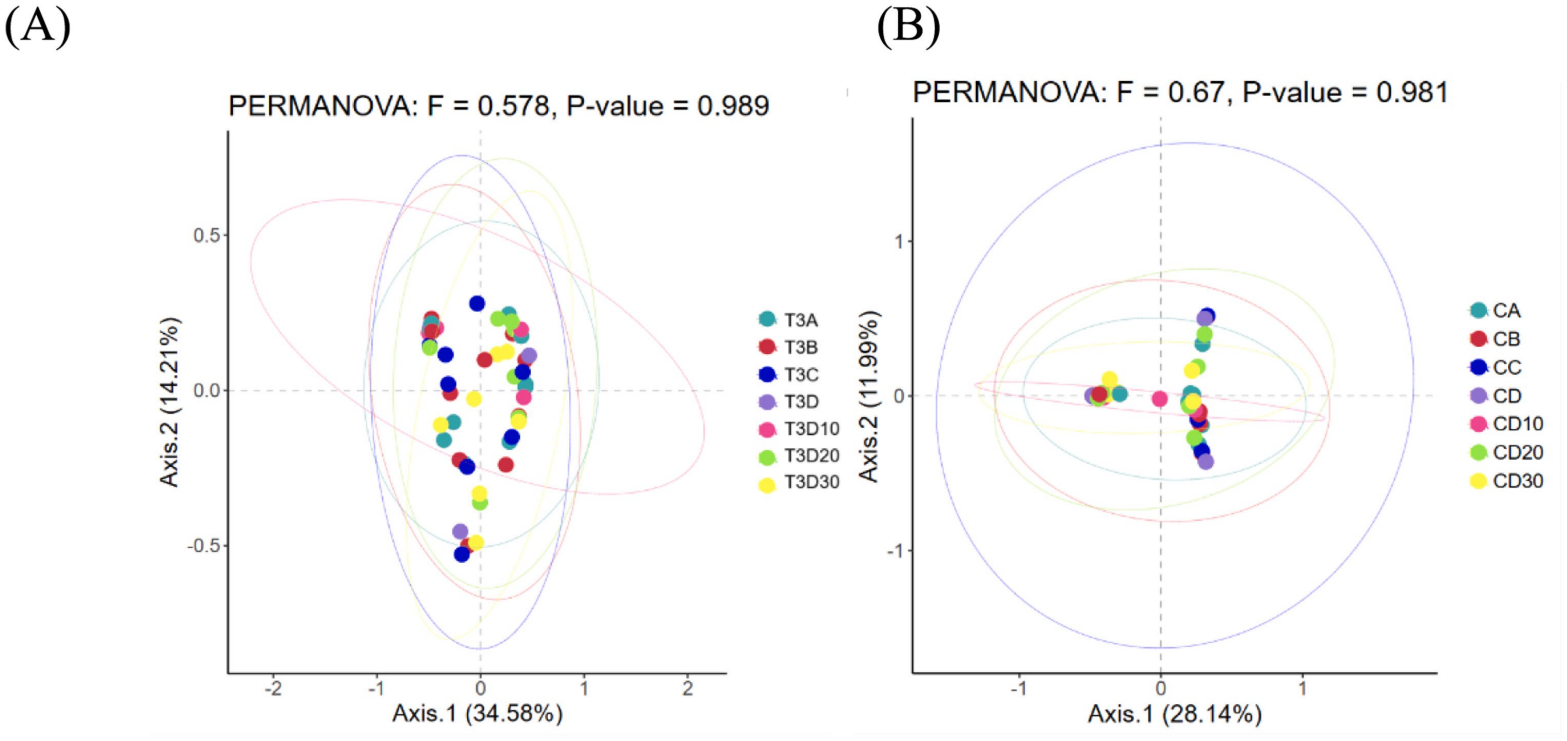
**(A)** Pre- and post-phototherapy changes in the microbiota of the experimental group. **(B)** Pre- and post-phototherapy changes in the microbiota of the control group.

**Figure 4:**
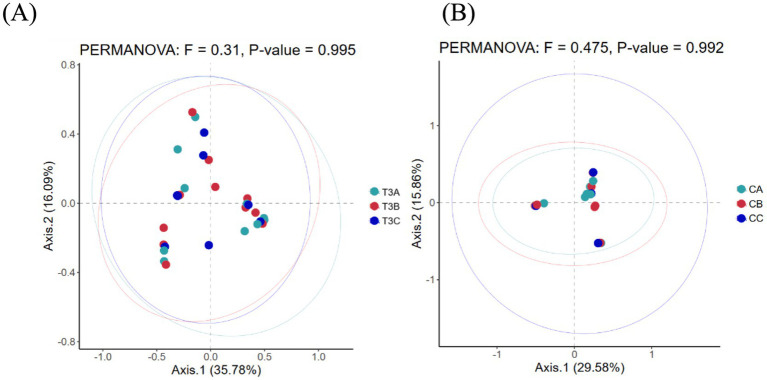
**(A)** Fluctuations in microbial composition within the experimental group before phototherapy and after 12 and 24 h of phototherapy. **(B)** Fluctuations in microbial composition within the control group before phototherapy and after 12 and 24 h of phototherapy.

**Figure 5:**
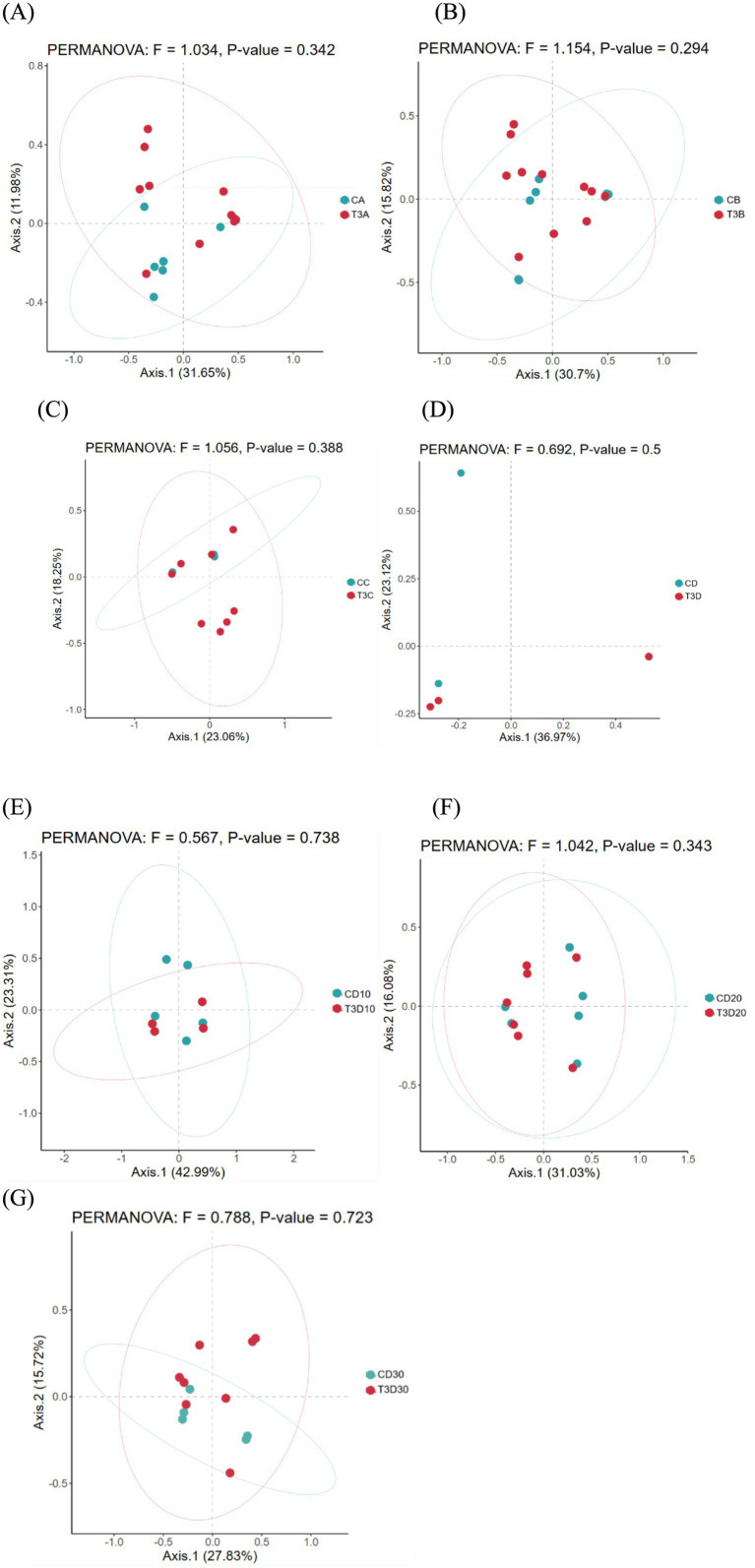
Comparison of gut microbiota between both groups before phototherapy **(A)**, after 12 **(B)**, 24 **(C)** and 36 h **(D)** of phototherapy, as well as 10 **(E)**, 20 **(F)** and 30 days **(G)** after delivery.

As a non-constrained method for dimensionality reduction, principal co-ordinates analysis (PCoA) was employed to investigate dissimilarities among samples. Both experimental and control groups were not statistically significantly different in microbial community shifts before and after phototherapy (*p* = 0.989, *p* = 0.981) (Figures 3A,B). Further analysis revealed that both groups were not statistically significantly different in microbial dynamics before phototherapy and after 12 and 24 h of phototherapy (*p* = 0.995, *p* = 0.992) (Figures 4A,B). Comparative analysis between experimental and control groups at corresponding times exhibited no statistically significant differences in microbial shifts before phototherapy, after 12, 24, and 36 h of phototherapy, and at the 10-, 20-, and 30-day follow-up (*p* = 0.342) (Figures 5A–G).

#### 3.2.4 Inter-group analysis at family, genus and species levels

**Figure 6:**
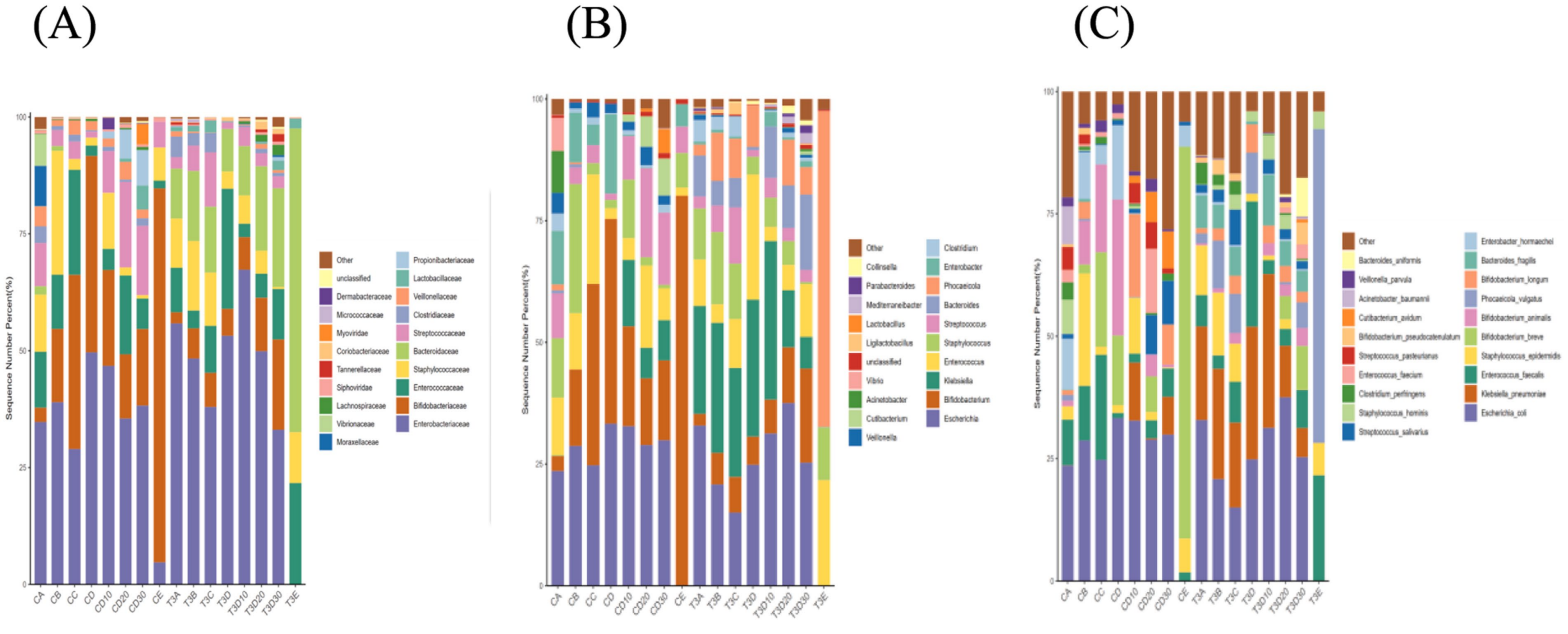
Bar chart illustrating the relative distribution of both groups at family **(A)**, genus **(B)**, and species **(C)** levels.

Analyses were conducted for both groups at family, genus and species levels, to more deeply elucidate the bacterial variations resulting from phototherapy and between both groups (Figures 6A–C).

At the family level, the dominant families in experimental and control groups were *Enterobacteriaceae*, *Bifidobacteriaceae*, *Staphylococcaceae*, *Enterococcaceae* and *Streptococcaceae*, and accounted for over 70 and 60% in experimental and control groups, respectively. The *Lactobacillaceae* levels of both experimental and control groups significantly increased after 12 h of phototherapy compared to before phototherapy. After phototherapy, the control group exhibited changes exceeding 1% in seven families: *Enterobacteriaceae*, *Bifidobacteriaceae*, *Enterococcaceae*, *Staphylococcaceae*, *Bacteroidaceae*, *Streptococcaceae* and *Veillonellaceae*. The experimental group showed changes in seven families, namely *Enterobacteriaceae*, *Bifidobacteriaceae*, *Enterococcaceae*, *Staphylococcaceae*, *Bacteroidaceae*, *Streptococcaceae* and *Clostridiaceae*.

At the level of genera, the dominant genera in both groups after phototherapy were *Escherichia*, *Bifidobacterium*, *Klebsiella*, *Staphylococcus* and *Enterococcus*, and constituted over 50 and 60% in experimental and control groups, respectively. Both groups showed a noticeable increase in Lactobacillus levels after 12 h of phototherapy compared to before phototherapy. After phototherapy, the experimental group exhibited changes exceeding 1% in nine genera: *Escherichia*, *Bifidobacterium*, *Klebsiella*, *Enterococcus*, *Staphylococcus*, *Streptococcus*, *Bacteroides*, *Phocaeicola* and *Clostridium*. The control group showed changes in nine genera, namely *Escherichia*, *Bifidobacterium*, *Staphylococcus*, *Streptococcus*, *Clostridium*, *Veillonella*, *Acinetobacter* and *Vibrio*.

At the level of species, *Escherichia coli* remained the most abundant species in both groups before and after phototherapy. After phototherapy, the experimental group exhibited changes exceeding 1% in nine species. To be specific, *E. coli*, *Klebsiella pneumoniae*, *Staphylococcus epidermidis*, *Bacteroides fragilis,* and *Clostridium perfringens* showed an increase, while *Enterococcus faecalis*, *Phocaeicola vulgatus*, *Bifidobacterium longum* and *Bifidobacterium pseudocatenulatum* demonstrated a decrease. The control group showed changes in 19 species. To be precise, a decrease occurred in *E. coli*, *E. faecalis*, *Bifidobacterium breve*, *Bifidobacterium animalis*, *Cutibacterium avidum*, *Veillonella parvula,* and *K. pneumoniae*; an increase took place in *S. epidermidis*, *Phocaeicola vulgatus*, *B. longum*, *Enterobacter hormaechei*, *Streptococcus salivarius*, *Staphylococcus hominis*, *Clostridium perfringens*, *Enterococcus faecium*, *Streptococcus pasteurianus*, *B. pseudocatenulatum*, *Acinetobacter baumannii,* and *Bacteroides uniformis*. Phototherapy may have a lesser impact on the changes in the gut microbiota of the experimental group.

#### 3.2.5 Linear discriminant analysis effect size analysis

**Figure 7:**
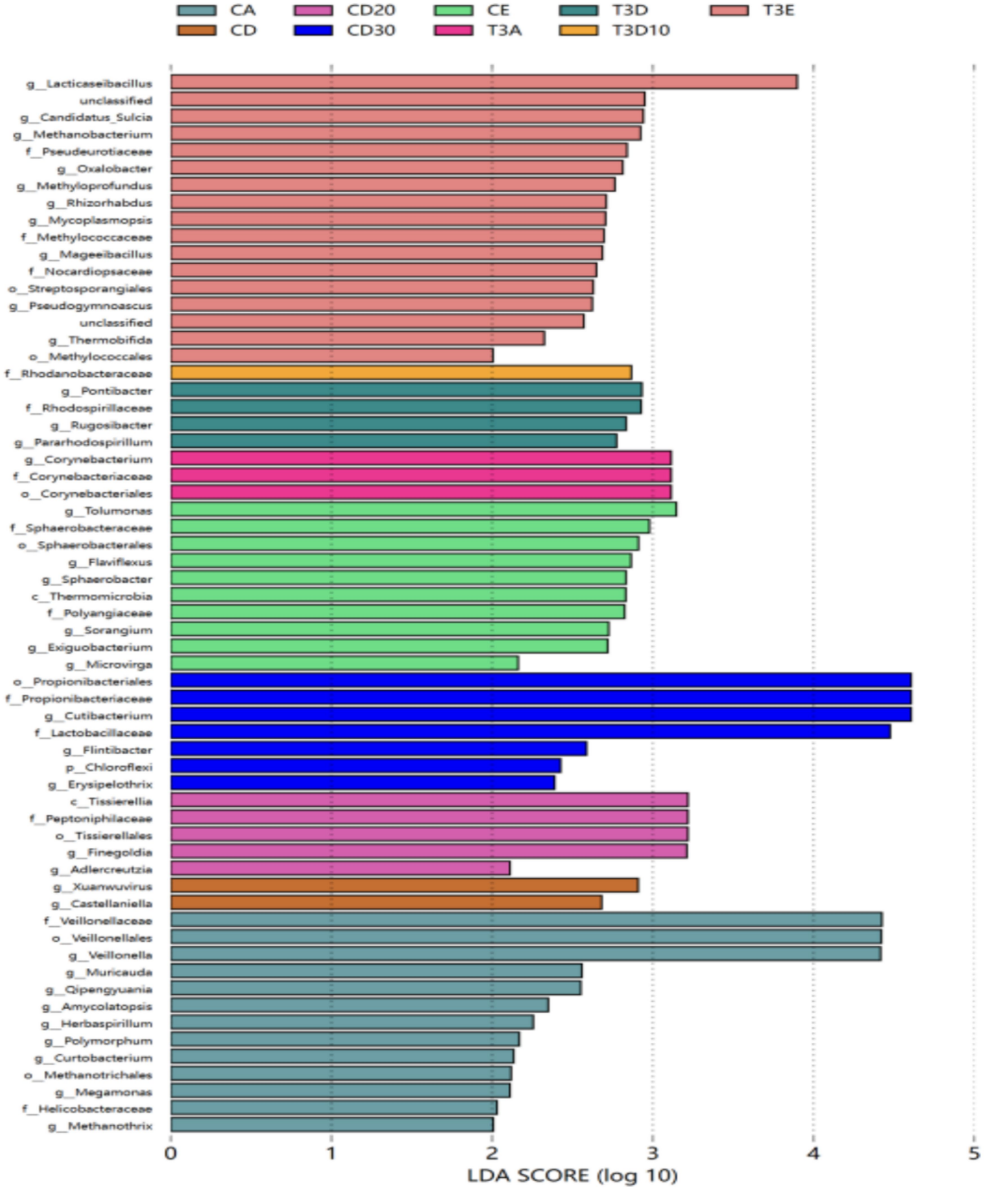
LEfSe analysis of LDA bar plots.

Linear discriminant analysis (LDA) effect size (LefSe) analysis identifies species with differential abundances among multiple groups and is typically utilized in biomarker research. As depicted in the LEfSe plot (Figure 7), 24 and 37 species significantly differing in abundance were identified at the genus level between experimental and control groups, respectively. Among these species, seven in the control group exhibited an LDA score greater than 4: *Propionibacteriaceae*, *Propionibacteriales*, *Cutibacterium*, *Lactobacillaceae*, *Veillonella*, *Veillonellales,* and *Veillonellaceae*. No species with significant differences were detected in the experimental group.

### 3.3 Metabolomic analysis

#### 3.3.1 OPLS-DA analysis

**Figure 8:**
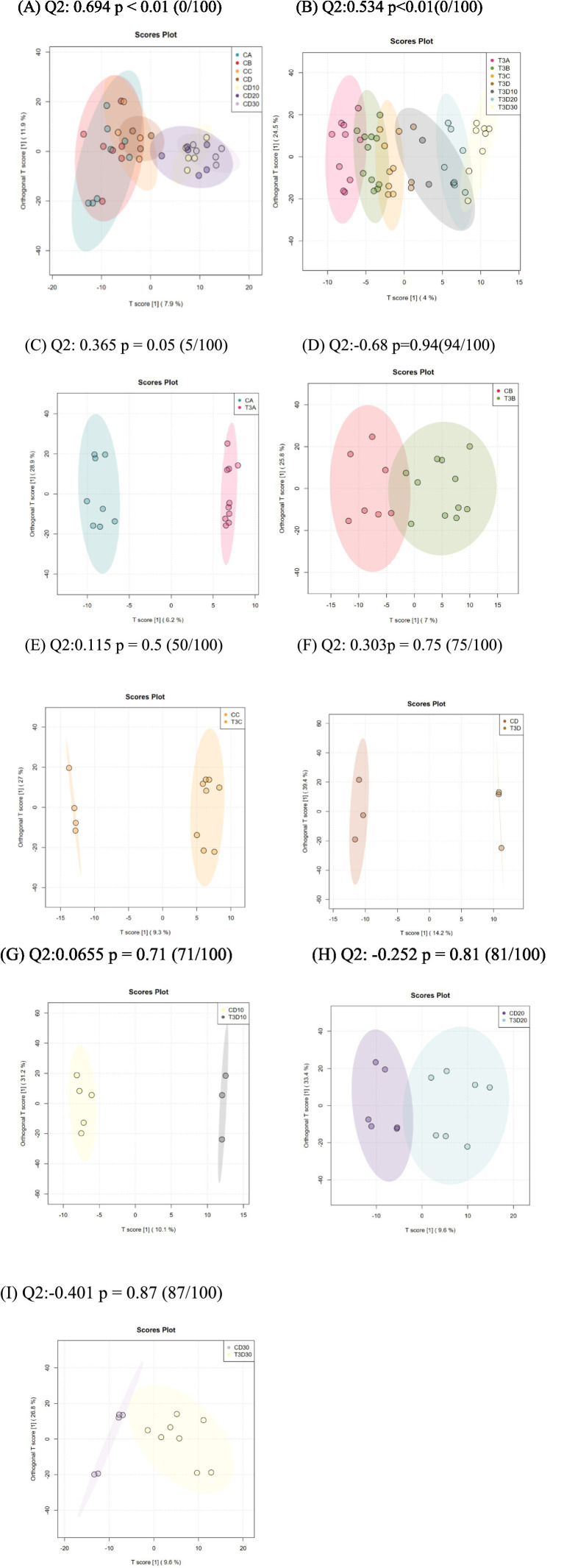
**(A)** Analysis of OPLSDA map in the experimental group at different times. **(B)** Analysis of OPLSDA map in the control group at different times. **(C–I)** Comparison of OPLSDA map between experimental group and control group before phototherapy **(C)**, after 12 **(D)**, 24 **(E)**, and 36 h **(F)** of phototherapy, as well as 10 **(G)**, 20 **(H)**, and 30 days **(I)** after delivery.

Orthogonal partial least squares discriminant analysis (OPLS-DA) is commonly employed to identify metabolites exhibiting significant between-group differences. In the permutation test of OPLS-DA, the Q2 statistic was utilized as the test metric and defined as Q2 = 1 – (model error variance/total model variance). The *p*-value was employed to assess whether the model showed significance, while Q2 quantified the predictive capability of the model. Values closer to 1 indicated superior predictive performance. Significant differential metabolites were observed both experimental and phototherapy groups before and after treatment (*p* < 0.01). However, no significant differential metabolites were identified in the concurrent comparison between experimental and control groups (Figures 8A–I).

**Figure 9:**
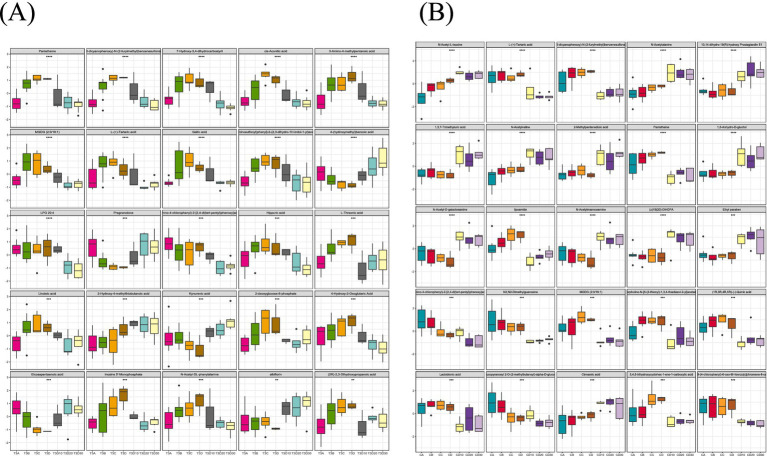
**(A)** A total of 25 metabolites with the largest statistical difference in the experimental group. **(B)** A total of 25 metabolites with the largest statistical difference in the control group.

The magnitude of metabolic changes is evaluated through the computation of fold changes (FCs), which, in conjunction with *p*-values, aids in the identification of metabolites meriting specific attention. For the visual illustration of between-group disparities, box plots (depicted in Figures 9A,B) were generated for the top-ranking, representative differentially expressed metabolites, as determined by univariate statistical analysis (limited to the top 25 with the smallest *p*-values).

#### 3.3.2 KEGG enrichment analysis

**Figure 10:**
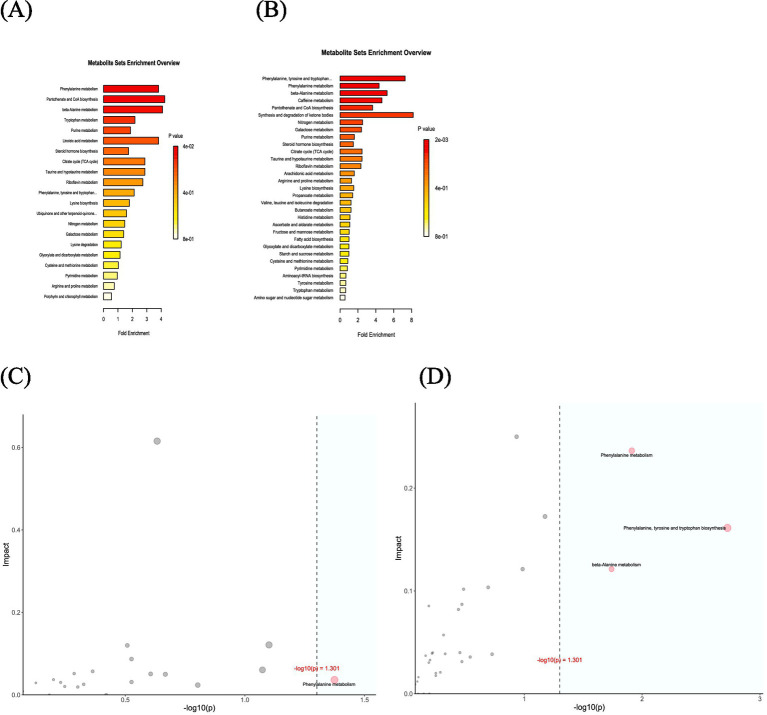
**(A)** Map analysis of differential metabolite enrichment in the experimental group. **(B)** Map analysis of differential metabolite enrichment in the control group. **(C)** Over-representation analysis (ORA) enrichment and topology analyses in the experimental group. **(D)** ORA enrichment and topology analyses in the control group.

KEGG enrichment analysis aims to identify pathways that exhibit statistically significant overrepresentation in the context of differential gene expression in comparison to the entire genomic landscape. The experimental group enriched 21 metabolic pathways, whereas the control one enriched 31 metabolic pathways. This indicates that the experimental group had a less pronounced impact on metabolism than the control one (Figures 10A,B). In the experimental group, the phenylalanine metabolic pathway played a crucial role. In the control group, pathways critical to phenylalanine, tryptophan and tyrosine biosynthesis, as well as phenylalanine and β-alanine metabolism, exhibited significant importance (Figures 10C,D).

## 4 Discussion

Neonatal jaundice represents the most common neonatal ailment in clinical practice and demands close follow-up and treatment. In addition, severe neonatal jaundice can lead to irreversible brain damage among newborns, which necessitates the alert attention of pediatricians and parents to this symptom. Presently, phototherapy is the predominant treatment method for jaundiced neonates (5). Nonetheless, this approach may produce certain side effects (12, 13), which makes it equally crucial to mitigate these adverse effects. Multiple studies have corroborated that the inclusion of some probiotics can diminish phototherapy-induced side effects, curtail the duration of hospital stays, and hasten the resolution of jaundice (8, 9). The findings of this study indicate that the addition of *L. rhamnosus* AB-GG exhibited no statistically significant differences in reducing the duration of hospital stays, improving weight gain or alleviating phototherapy-induced side effects among jaundiced neonates. This disparity might stem from variations in the colonization capacities of different strains within neonatal intestines and the diversity of their primary metabolic products. The clinical effectiveness of *L. rhamnosus* in treating jaundice yields inconsistent outcomes. A prospective, double-blind and placebo-controlled trial that involved 60 neonates with hemolytic jaundice caused by isoimmunization demonstrated that patients receiving *L. rhamnosus* were not significantly different in serum total bilirubin levels within 24 h of birth or duration of phototherapy. However, the experimental group exhibited notably increased bowel movement frequency 48 and 72 h after delivery. In contrast to the control group, the experimental one demonstrated significantly less rebound in serum total bilirubin levels 36 h after delivery (9). Conversely, another study on the application of *L. rhamnosus* in neonatal jaundice revealed that the probiotic group experienced significantly reduced mean total bilirubin levels on days 3, 5, and 10, which was accompanied by a marked elevation in average bowel movement frequency. A negative correlation was found between the frequency of bowel movements and total bilirubin levels on days 3, 5, and 10, which suggested that immediate postnatal support with probiotics (*L. rhamnosus* GG) positively affects bilirubin metabolism and potentially lowers the risk of hyperbilirubinemia (14). In this study, a rising trend was observed despite no statistically significant differences in the average frequency of bowel movements between control and experimental groups, which is consistent with prior research findings.

Unconjugated bilirubin undergoes conjugation with glucuronic acid in the liver through the action of glucuronyltransferase to yield bilirubin monoglucuronide and diglucuronide. Soluble in water, bilirubin monoglucuronide and diglucuronide are secreted into bile by active transport and subsequently excreted into the small intestine if necessary (15). Upon birth, neonatal intestines exhibit reduced intestinal flora, with substantial bilirubin in the meconium (4). As an intestinal mucosal enzyme, β-glucuronidase is highly concentrated in preterm and term neonates, which catalyzes the conversion of conjugated bilirubin to unconjugated bilirubin and thereby facilitates its reabsorption from the gut (16). The increase in enterohepatic circulation further elevates the serum total bilirubin levels in neonates, with this cycle contributing to a rise of roughly 30% in serum bilirubin concentrations (17). The rapid colonization of the gastrointestinal tract by various bacteria after delivery is a crucial factor in reducing enterohepatic circulation in neonates (18). A decrease in microbial diversity, which is a primary feature of dysbiosis, can cause a variety of metabolic disorders. The primary mechanisms by which the gut microbiota exerts its effects involve direct or indirect modulation of the composition of the host’s intestinal microbiota, activation of endogenous microbial communities within the host, or stimulation of the host’s immune system. The reduction of these beneficial gut microbiota may lead to adverse effects associated with phototherapy (4, 7, 9). By analyzing the total number of gut microbiota in experimental and control groups, this study found that phototherapy reduces the total gut microbiota, which is in line with previous findings (19). Nevertheless, the gut microbiota gradually recovered as phototherapy ceased and infants grew. The experimental group was fully restored by the 30-day follow-up, whereas the control one was still in a decremental state. Analyzing the *α* diversity between the two groups revealed higher Shannon, Simpson, Observed and Chao1 indices in the experimental group, which indicated greater richness and evenness of gut microbiota. Nonetheless, β diversity analysis showed no significant differences between both groups before and after phototherapy. These results revealed that adding *L. rhamnosus* AB-GG appears to mitigate the detrimental effects of phototherapy on gut microbiota, and aids in its stabilization. The two groups demonstrated significantly increased Lactobacillus genera 12 h after phototherapy compared to baseline but showed no notable difference in the extent of the increase. This conforms to past studies highlighting Bifidobacterium as the most critical species for the development of gastrointestinal flora during the neonatal period, which markedly decreases following phototherapy in neonatal jaundice patients (20). Consequently, the addition of different probiotics is likely to exert varied effects on the gut microbiota of neonatal jaundice patients. *L. rhamnosus* may help maintain intestinal flora homeostasis instead of being the optimal strain for aiding neonatal jaundice patients. The immediate postnatal administration of probiotic microorganisms via the enteral route may support gastrointestinal colonization, which potentially facilitates the recovery of neonatal jaundice through the regulation of bacterial colonies, enhances gut motility, and reduces enterohepatic circulation (19). Upon the follow-up of the experimental and control groups at 7–14 days after discharge, stool frequency of experimental groups was decreased commonly. We observed *B. fragilis* showed an increase. The increase of *B. fragilis* is associated with children suffering from constipation (21). At the same time, *B. longum* which can promote intestinal peristalsis and inhibit the growth of harmful bacteria demonstrated a decrease (22). We hypothesize that these changes in gut microbiota observed in the experimental group led to a reduction in stool frequency during the follow-up period.

Through metabolite analysis, this study observed that the metabolic composition structures of both groups underwent changes before and after phototherapy. However, no significantly differential metabolites were identified when the groups were compared at different time points. The analysis and comparison of the fundamental metabolic pathways within the KEGG database suggested that the experimental group was enriched in 21 metabolic pathways, while the control one was enriched in 31 pathways. In both groups, differential metabolites played a key role in the phenylalanine metabolic pathway. Several studies indicate altered serum phenylalanine metabolism in neonates is closely associated with the presence of combined bilirubin encephalopathy in neonates with hyperbilirubinemia (18, 23). In the control group, differential metabolites also played a crucial role in the biosynthesis of phenylalanine, tyrosine and tryptophan, as well as the β-alanine metabolic pathway. β-alanine is a pivotal component for pantothenate synthesis and the precursor to coenzyme A, an essential vitamin for multiple biochemical processes (24). Prior research has uncovered a negative correlation between the 25-(OH)D_3_ levels and indirect bilirubin in infants with jaundice (25). Disturbances in gut microbiota may influence β-alanine metabolism, which thereby affects vitamin synthesis and ultimately the recovery of jaundiced newborns.

There are limitations in this study. Because stool frequency of experimental groups was decreased commonly during the follow-up period after discharge, we terminated the study in advance. This decision result in a limited sample size, which may contribute to no difference between experimental and control groups. However, the results in our study is consistent with prior research findings. We believe that our results are credible. We should improve the method of probiotic application by adjusting the probiotic content and changing the number of doses to increase the sample size in later studies.

## 5 Conclusion

The supplementation of *L. rhamnosus* AB-GG to experimental groups resulted in fewer stools during the follow-up period, leading us to terminate the study. Due to our study results we still observed *L. rhamnosus* supplementation was shown to mitigate intestinal dysbiosis in neonates with jaundice, which thereby facilitated a more rapid recovery of gut microbiota depleted by phototherapy. After discontinue administration of probiotics, stool frequency is normal in the experimental groups. This phenomenon indicated that the dosage and regimen of probiotic application require further investigation.

## Data Availability

The raw data supporting the conclusions of this article will be made available by the authors, without undue reservation.

## Footnotes

^1^ https://www.mzcloud.org/

^2^ https://www.genome.jp/kegg/pathway.html
