# Supplementary material for: Effect of Lactobacillus rhamnosus AB-GG combined with phototherapy on neonatal jaundice indicators, intestinal microbiota and metabolism
Source: Front Nutr. 2025 Apr 8;12:1581242. doi: 10.3389/fnut.2025.1581242 (PMC12011606; doi:10.3389/fnut.2025.1581242)
Supplement: Supplementary file 1 [file Table_1.DOCX]

# CONSERVE Checklists

Use CONSERVE-CONSORT for completed trial reports and CONSERVE-SPIRIT for trial protocols.

| CONSERVE-CONSORT Extension: [DATE] | | | | | |
| --- | --- | --- | --- | --- | --- |
| Item | Item Title | Description | | | Page No. |
| I. | Extenuating Circumstances | Describe the circumstances and how they constitute extenuating circumstances. | | |  |
| II. | Important Modifications | 1. Describe how the modifications are important modifications. | | |  |
|  |  | 1. Describe the impacts and mitigating strategies, including their rationale and implications for the trial. | | | (see below) |
|  |  | 1. Provide a modification timeline. | | |  |
| III. | Responsible Parties | State who planned, reviewed and approved the modifications. | | |  |
| IV. | Interim data | If modifications were informed by trial data, describe how the interim data were used, including whether they were examined by study group, and whether the individuals reviewing the data were blinded to the treatment allocation. | | |  |
| CONSORT Number and Item | | For each row, if important modifications occurred check “direct impact” and/or “mitigating strategy” and describe the changes in the trial manuscript or supplement. Check “no change” for items that are unaffected in the extenuating circumstance. | | | Page No. |
|  |  | No Change | Impact* | Mitigating Strategy** |  |
| 1 | Title and abstract |  | In our registration study(Registration No.: ChiCTR2000036013), three probiotics were utilized. This manuscript is only about Lactobacillus rhamnosus AB-GG, which is one of the three probiotics. |  |  |
| 2 | Introduction | √ |  |  |  |
| 3 | Methods: Trial Design | √ |  |  |  |
| 4 | Methods: Participants |  | In the protocol, there are both full term infants and preterm infants in this study. Our manuscript just reports the result of full term infants. |  |  |
| 5 | Methods: Interventions | √ |  |  |  |
| 6 | Methods: Outcomes | √ |  |  |  |
| 7 | Methods: Sample Size |  |  | Upon the follow-up of the experimental and control groups at 7-14 days after discharge, stool frequency of experimental groups was decreased commonly. To avoid the parental panic for this phenomenon, we decided to stop the supplementation of Lactobacillus rhamnosus AB-GG. About three days after stopping probiotics, stool frequency is normal in the experimental groups. However, this study was still prematurely terminated. |  |
| 8-10 | Methods: Randomisation | √ |  |  |  |
| 11 | Methods: Blinding | √ |  |  |  |
| 12 | Methods: Statistical methods | √ |  |  |  |
| 13 | Results: Participant flow | √ |  |  |  |
| 14 | Results: Recruitment | √ |  |  |  |
| 15 | Results: Baseline data | √ |  |  |  |
| 16 | Results: Numbers analysed | √ |  |  |  |
| 17 | Results: Outcomes and estimation | √ |  |  |  |
| 18 | Results: Ancillary analyses | √ |  |  |  |
| 19 | Results: Harms | √ |  |  |  |
| 20 | Discussion: Limitations | √ |  |  |  |
| 21 | Discussion: Generalisability | √ |  |  |  |
| 23 | Other information: Registration | √ |  |  |  |
| 24 | Other information: Protocol | √ |  |  |  |
| 25 | Other information: Funding | √ |  |  |  |
| *Aspects of the trial that are directly affected or changed by the extenuating circumstance and are not under the control of investigators, sponsor or funder.  **Aspects of the trial that are modified by the study investigators, sponsor or funder to respond to the extenuating circumstance or manage the direct impacts on the trial. | | | | | |

| CONSERVE-SPIRIT Extension: [DATE] | | | | | |
| --- | --- | --- | --- | --- | --- |
| Item | Item Title | Description | | | Page No. |
| I. | Extenuating Circumstances | Describe the circumstances and how they constitute extenuating circumstances. | | |  |
| II. | Important Modifications | 1. Describe how the modifications are important modifications. | | |  |
|  |  | 1. Describe the impacts and mitigating strategies, including their rationale and implications for the trial. | | | (see below) |
|  |  | 1. Provide a modification timeline. | | |  |
| III. | Responsible Parties | State who planned, reviewed and approved the modifications. | | |  |
| IV. | Interim data | If modifications were informed by trial data, describe how the interim data were used, including whether they were examined by study group, and whether the individuals reviewing the data were blinded to the treatment allocation. | | |  |
| SPIRIT Item and Number | | For each row, if important modifications occurred, check one or both of “impact” and/or “mitigating strategy” and describe the changes in the protocol. Check “no change” for items that are unaffected in the extenuating circumstance. | | | Page No. |
|  |  | No Change | Impact* | Mitigating Strategy** |  |
| 1 | Title | √ |  |  |  |
| 2 | Trial registration | √ |  |  |  |
| 3 | Protocol version | √ |  |  |  |
| 4 | Funding | √ |  |  |  |
| 5 | Roles and responsibilities | √ |  |  |  |
| 6 | Background and rationale | √ |  |  |  |
| 7 | Objectives | √ |  |  |  |
| 8 | Trial design | √ |  |  |  |
| 9 | Study setting | √ |  |  |  |
| 10 | Eligibility criteria | √ |  |  |  |
| 11 | Interventions | √ |  |  |  |
| 12 | Outcomes | √ |  |  |  |
| 13 | Participant timeline | √ |  |  |  |
| 14 | Sample size | √ |  |  |  |
| 15 | Recruitment | √ |  |  |  |
| 16 | Allocation | √ |  |  |  |
| 17 | Blinding (masking) | √ |  |  |  |
| 18 | Data collection methods | √ |  |  |  |
| 19 | Data management | √ |  |  |  |
| 20 | Statistical methods | √ |  |  |  |
| 21 | Data monitoring | √ |  |  |  |
| 22 | Harms | √ |  |  |  |
| 23 | Auditing | √ |  |  |  |
| 24 | Research ethics approval | √ |  |  |  |
| 25 | Protocol amendments | √ |  |  |  |
| 26 | Consent or assent | √ |  |  |  |
| 27 | Confidentiality | √ |  |  |  |
| 28 | Declaration of interests | √ |  |  |  |
| 29 | Access to data | √ |  |  |  |
| 30 | Ancillary and post-trial care | √ |  |  |  |
| 31 | Dissemination policy | √ |  |  |  |
| 32 | Informed consent materials | √ |  |  |  |
| 33 | Biological specimens | √ |  |  |  |
| *Aspects of the trial that are directly affected or changed by the extenuating circumstance and are not under the control of investigators, sponsor or funder.  **Aspects of the trial that are modified by the study investigators, sponsor or funder to respond to the extenuating circumstance or manage the direct impacts on the trial. | | | | | |
